# Exploring the Experience of Nursing Home Residents Participation in a Hope-Focused Group

**DOI:** 10.1155/2014/623082

**Published:** 2014-01-16

**Authors:** Sharon L. Moore, Susan E. Hall, Jennifer Jackson

**Affiliations:** ^1^Faculty of Health Disciplines, Athabasca University, 1 University Drive, Athabasca, AB, Canada T9S 3A3; ^2^AgeCare, Beverly Centre Glenmore, 1729 90 Avenue, SW, Calgary, Canada T2V 4S1; ^3^Nursing Professional Practice Department, The Ottawa Hospital AB, Central Campus, 501 Smyth Road, Ottawa, ON, Canada K1H 8L6

## Abstract

A qualitative intervention was used to explore how older adults living in a long-term care environment (nursing home) understand hope and experience being participants in a group in which a hope intervention was carried out. A group project in which each session focused intentionally on a hope strategy was carried out with a convenience sample of 10 women (ages 75–99) who were members of an existing group. Data were analyzed using thematic analysis of the interviews (conducted before the group intervention was carried out and again at the end), field notes, and collaborative conversations regarding emerging themes. Findings from this study suggest that hope is not static and that it can change over time in response to one's situations and circumstances. Also evident in this study is the potential for using a group process in long-term care to foster hope in an intentional way to make it more visible in the lives of the residents and their environment suggesting that one is “never too old for hope.”

## 1. Introduction

Hope has an enduring ability to influence the health and wellbeing of individuals across the lifespan. It is described as a key psychosocial resource that helps individuals through a variety of difficult circumstances in their lives [1–5]. Further, hope has been identified as an important ingredient in fostering a sense of meaning and purpose in life and enhancing quality of life for older adults [6, 7]. As people age, they frequently experience physical and psychological challenges and losses in their lives and for some of these individuals, these losses lead to placement in nursing homes and long-term care. Transitioning to long-term care can result in further losses increasing older persons' vulnerability to illness, depression, and loss of hope [8]. Therefore, finding ways to foster hope for older adults in long-term care is essential. For purposes of this paper, long-term care is defined as residential care which provides full-time care to individuals (generally older adults) and is also often referred to as nursing home care.

Nurses, regardless of their clinical setting, have an opportunity to instill hope in clients and to appreciate its significance in sustaining both their own personal mental health and that of their clients/patients [5, 9, 10]. Given the proximity and frequency of contact with patients, nurses who work in nursing homes in long-term care are in key positions to instill and foster hope in residents. While the importance of hope is often acknowledged, ways to explicitly foster its development have not always been clear. By examining the role of nursing interventions in the development of hope, it may be possible to identify ways in which nurses can assist residents in creating or nurturing hope in their own lives. The purpose of this paper is to describe a study that intentionally used hope as an intervention in a group setting in long-term care.

## 2. Background

Hope has been defined as an important concept for seniors across many cultures [7, 11–13]. The understanding of hope encompasses a holistic, multidimensional construct [14, 15]. Yet how one finds hope when hope is gone, or how to facilitate the development of hope is still somewhat elusive.

There are a growing number of studies that examine aspects of hope and aging. These studies support the significance of hope in many areas related to the lives of older adults. After interviewing 10 seniors with early-stage dementia who described their experiences of hope, Wolverson et al. [16] concluded that hope was a developmental construct associated with experiences throughout the participants' lives. They described hope as a dynamic, relational aspect integral to the daily lives of the participants. It is notable that hope was not focused on recovery from illness; rather, participants hoped for good outcomes for loved ones and for society as well. Transcending individual hope in this way was reported as a unique view of hope for the future as a legacy of hope.

Duggleby [17] described ways to foster hope with incarcerated older adults. She identified strategies for helping to foster hope in seniors. Examples of these strategies were creating opportunities to help seniors have some control in their lives, facilitating seniors to set goals and make decisions related to their care, using life review techniques to help seniors make meaning of their lives and exploring with seniors, ways in which they might leave a legacy. These hope-fostering strategies are consistent with those identified in palliative care literature [3].

A grounded theory study showed an emerging theory of “renewing everyday hope” in which family caregivers of persons living with dementia found strength in relying on hope as they cared for their loved ones, suggesting the importance of health professionals paying attention to and learning more about how to foster hope [18].

There is a dearth of literature exploring hope in the context of group-based interventions. One study found that a hope-based intervention was not successful at increasing hope for depressed seniors [19]. However, other studies have been encouraging. Pretest/posttest interventions demonstrated increased hope following participation in a facilitated group [20, 21]. The first of these studies was a six-session hope intervention offered in a group psychotherapy program for individuals undergoing genetic testing for hereditary colorectal cancer. The group sessions focused on a discussion of hope theory, setting goals, hope pathways (“skills and strategies to achieve goals”), and hope agency (“energy and motivation to achieve goals”) [20]. The second study demonstrated an increase in positive affect and decrease in loneliness for widowed seniors who participated in a support group [21]. Another study, designed to implement and evaluate a hope intervention program in a group of individuals with a first recurrence of cancer, found that the people participating in the hope group had a significantly higher mean score on a hope scale (Herth Hope Index) than participants in the information and control groups [22]. Herth [22] concluded that it is possible for nurses to “increase the feeling of hope and perceived quality of life in individuals with first recurrence of cancer through specifically designed hope interventions” [22]. While these studies add to an understanding of hope interventions, there is a need for research carried out in a long-term care environment.

Understanding the effectiveness of strategies aimed to enhance hope in older adults in long-term care is critical in order for nurses to influence the development of hope for the residents they care for. This project adds to a growing body of literature on how nurses and health care professionals can foster and enhance hope in long-term care through the implementation of a hope-focused group program.

## 3. The Study 

### 3.1. Aims

Given the limited research relating to hope-focused intervention with seniors, this project aimed to contribute to an understanding of how hope may become more visible in long-term care through a group setting. Given that hope is believed to be strengthened in community, a group setting was a logical context in which to introduce hope-focused sessions.

The purpose of this project was to foster the intentional use of hope for residents in a nursing home through introducing and implementing a hope-focused curriculum in an ongoing group program. Further, the project aimed to deepen the understanding of how older adults understand the concept of hope and how they experience it through the group process.

The researchers implemented the project with a strong conviction that hope is a necessary element for seniors to achieve and maintain a good quality of life. Three key aspects of the construct of hope guided this intervention and were the basis of the design and working assumptions of the program delivery. They were as follows: (1) hope is about envisioning a future in which older residents in long-term care would be willing to participate, even in the face of adverse circumstances; (2) residents would be more willing to try things and risk participating in the programs offered; and (3) without hope, residents might feel at the mercy of circumstances and slip into depression or despondency.

### 3.2. Design

This qualitative intervention study intended to explore the research question “what is the experience of older adults participating in a hope-focused group?” The study consisted of a preinterview with residents of a long-term care facility to explore their understanding of hope, a 9-session “group hope intervention,” followed by a postinterview that explored the meaning and experience of participating in the group. This study employed a qualitative descriptive research design as defined by Sandelowski [23]. Sandelowski [23] explains qualitative descriptive studies as providing a comprehensive summary of events in the everyday terms of those events. Further, Sandelowski [23] notes that “researchers conducting such studies seek an accurate accounting of events that most people observing the event would agree is accurate… and an accurate accounting of the meanings participants attributed to those events that those participants would agree is accurate ” (p. 336).

### 3.3. Ethical Considerations

The study received ethical approval at the researcher's home university and at the ethics approval board at the long-term care centre. This population is potentially vulnerable to health status changes; thus, the group leaders were careful to maintain vigilance to any health changes (physical and mental) that might influence residents' wellbeing and, or ability to participate.

### 3.4. Participants

A purposive, convenience sample was used for this study. The members (all women) of a regularly scheduled preexisting support group (the Good Grief Coffee Club) led by the two researchers were approached and invited to participate in the study (this group started several years prior as a grief support group for women who had lost a spouse. Over the years, it evolved into more of a support/conversation group which meets twice a month). The voluntary nature of participation was explained and involvement in a different group was offered to anyone who did not wish to participate. All of the women (ranging in age from 75 to 99) in the group agreed to take part. Their expressed motivation was that if they could help someone else, they would be “happy to do so.” For the time frame of the study, the group was kept as a closed group (meaning no new members were admitted) in order to keep the study data within the same group of participants.  Table 1 identifies demographic characteristics of the group participants.

### 3.5. Data Collection

A university professor who is a registered nurse and registered psychologist and the pastoral care nurse (who had been working together in an ongoing group that meets twice per month) partnered to complete this research project. The pastoral care nurse brought knowledge of the group and group process and familiarity with the environment of the nursing home, while the professor brought an understanding of hope and strategies for fostering hope. Both researchers have had extensive education and experience in working with older adults across a variety of settings as well as educational preparation in working with groups.

The participants were interviewed prior to start of the hope-focused group sessions to ascertain their understanding of the concept of hope and again at the end of the group sessions to ask them to reflect on their experience of being in the group. These interviews were audio-recorded. Throughout each group session, one of the group leaders took responsibility for writing field notes which included the focus of the session, participants in attendance, key observations on the group process, key phrases, and points made by the participants. Following each session, the two group leaders met to debrief, review the session, and share their reflections and perceptions with each other. Notes from these debriefing sessions were also included as part of the field notes and were transcribed for purposes of analysis. The understandings and meanings that participants shared within the group were confirmed and verified (and recorded as field notes) throughout each group session.

### 3.6. The Intervention

The organizing principle for this study was HOPE and the belief that having hope contributes to quality, meaning, and purpose in life. The book *Finding Hope: Ways to See Life in a Brighter Light *was selected as a “textbook” for the group. This small book contains short one-page strategies that the authors [24] developed. Each resident was provided with a copy of the book. This book was chosen primarily because it was well known to the group leaders, it was small and inexpensive, and it contained very practical and doable strategies. Nine topics from the *Finding Hope* book were selected to provide a focus for group sessions. While the group leaders identified the topics ahead of time, it is important to note that they were open to an *emerging design* based on postsession reflections of group responses and needs on an ongoing basis.

The objectives of the group were to

- use hope intentionally through a small group program for residents in long-term care;
- assess the impact of participating on the program from the residents' perspectives;
- develop a facilitator's guide as a prototype for use in other long-term care settings.

The group met twice per month over a 5-month period. The researchers interviewed participants before the group sessions started and again at the end of the group sessions to ascertain their perceptions of hope before and after the group and to have them talk about their own experience as a group member. They interviewed four participants a third time to clarify their experience of participation in the group. Each group session followed a consistent format as follows:

- orientation (reorientation) to the project;
- a review of ethics and the option to participate or not;
- brief recap of the previous session;
- reading of the current “hope strategy” (group members took turns reading the strategy each week);
- questions/discussion about the hope strategy;
- summary and “homework” for the next session.

The initial plan had been for one of the group leaders to read the “hope strategy” for the session but in asking the group, there was always a volunteer from the group to do this. This served several purposes. It gave the opportunity for participation to the group members; it reoriented all of the group (and leaders) to the assigned homework and task for the current session. Following the reading, one of the group leaders initiated the discussion by asking general questions about hope based on the reading. Group members had the option to participate or not in the discussion but were asked specifically if they had anything they would like to add. Very frequently, people did not wait to be asked but would offer their ideas. At the close of each session, one of the group leaders announced the homework for the following session (to read one page from the *Finding Hope* book) in preparation for the next session.

The group session topics focused on exploring the concept of hope, noticing signs of hope, listening to the voice of hope, looking back at personal experiences of hope, finding hope in unexpected places, borrowing hope, learning to tilt, making a small difference, and celebrating hope.  Table 2 provides an outline of these topics and the questions used to facilitate the hope discussions.

In the final session on celebrating hope, the group leaders wanted to express their thanks and gratitude to the group members for helping them in this project by doing something that Jevne and Miller [24] described in their book as “Celebrate Hope.” “When you celebrate, you communicate something is important enough to be highlighted or someone is valued enough to be fussed over. It is a way of showing gratitude and appreciation. It is a way of showing love” (p. 51). For this session, they hosted a catered luncheon in honour of these residents who had committed to offering their perspectives that could be shared with others. The lunch was set up in a separate room from their normal dining room (with tablecloths and fancy napkins to add to the ambiance). To reinforce that the residents were important and valued, the leaders read the “celebrating hope” strategy, reinforcing the significance of their contributions to our understanding of hope in the group process. The residents talked about how they valued being recognized in this way and frequently commented (both during and after the lunch) on how much they enjoyed the lunch and having an opportunity to celebrate in this way.

### 3.7. Data Analysis

Following the completion of the group sessions, a research assistant (a graduate student) joined the research team to assist with the literature review and analysis. The data were analyzed thematically drawing on processes described by Creswell [25] and Glesne [26]. The analysis process involved organizing and categorizing the data. The audio interviews before and after group were transcribed by a transcriptionist who was asked to sign a confidentiality pledge as an added measure of protecting the confidentiality of the participants. The interview transcriptions along with the transcribed field notes provided data sources. The transcripts were read and reviewed separately by each of the two researchers and were coded for key words and statements. Using a process of collaborative analysis described by van Manen [27], the researchers along with the research assistant met together for a series of collaborative conversations to discuss themes that evolved and key findings. Throughout this process, the researchers kept track of insights and decisions through memo writing, as analytic thoughts occurred either individually or as part of the collaborative conversations. Insights from each set of transcripts, the field notes, the analytic memos, and the collaborative conversations served to deepen understanding of the research question.

## 4. Findings

Thematic analysis revealed three key themes from the preintervention interviews and five themes from the experience of participating in the group. These are reported separately because the main focus of the preintervention interviews was to develop an understanding of how the group participants understood hope, while the post interview focus was to learn about their experiences of participating in a hope-focused group.

### 4.1. Themes from Preintervention Interviews

Three themes that represented the participants' perception of hope emerged from the preintervention interviews including: (1) hope as future; (2) hope as acceptance; and (3) hope as fuel.

#### 4.1.1. Theme 1: Hope as Future

Even though the participants were in the later stages of life, they still talked about hope for a better tomorrow. They accepted where they were at in their stage of life but also maintained a belief in the possibility that things could be better tomorrow. As one participant stated “I think everybody's got a little bit of hope in them that they want to look for the next day or see what's going to happen.” It was clear that some of the participants described hope for the future in the context of a challenging present, believing that things had to be better in the future.

#### 4.1.2. Theme 2: Hope as Acceptance

 Participants talked about hope as accepting their circumstances. They portrayed hope as a realistic hope, a hope in which they accepted their circumstances as illustrated in the words of three participants:

*“Somehow I have to find a balance between what I can do and what I can't do and I don't know how you do that. You just do it.”*

*“It could always be worse.”*

*“In fact, you know, you do not hope for very much at this stage of the game. You just hope that you can contribute a little bit to people.”*

All of the participants are living in a nursing home environment and it is quite likely that they will spend their remaining days there. Even though many of them would desire to be living in their own homes, they accept the reality of being where they are. For many of them, hope was what helped them make the transition to accepting their present circumstances and to experience a sense of home. This was reflected in the words of one participant who said “you should not hope for what you cannot have.”

#### 4.1.3. Theme 3: Hope as Fuel

Participants described hope as providing the energy and encouragement to keep going in the face of adversity. In some cases, this energy of hope was linked to survival. These thoughts were poignantly illustrated in the words of the participants:

*“You have to hope no matter what. I never give up. I don't know what there is for me in life left, but I never gave in.”*

*“Hope keeps you alive. You are in dire circumstances and all we had was hope. It keeps you going… I will survive I said to myself. I got not to give in. I got to survive.”*

Hope was described as the difference between getting up and trying again and literally giving up and dying. For example, some participants faced great adversity such as the death of a child by suicide, living through wars and loss of significant others, and the resulting disruption of everything they knew. Hope provided the fuel to keep on going even when they felt like giving up.

### 4.2. Themes from Experiences of Being in the Group

Five key themes emerged from the analysis of participants' experiences of being in the group and what it was like to participate in the group sessions that were focused on hope. These themes arose from our observations throughout the group sessions, follow-up interviews with the participants and the collaborative conversations about the findings. The themes included (1) building a sense of community; (2) giving and receiving support; (3) normalizing experience; (4) developing a more positive perspective; and (5) thinking more intentionally about hope.

#### 4.2.1. Theme 1: Building a Sense of Community

On several occasions during the group sessions and the follow-up interviews, the participants spoke about how they enjoyed being part of the group:

*“I like the group. I think it's great to get together and hear stories. They've all got a story.”*

*“I enjoyed that group. The fellowship I suppose.”*

This reflects a sense of togetherness as a result of being in the group and suggests that hope may be strengthened in community. It appears that hope experienced in this group setting became more visible, more real for people because when hope was low for one person, others “came to the rescue” to encourage and support one another.

#### 4.2.2. Theme 2: Giving and Receiving Support

The giving and receiving of support was obvious on many occasions throughout the group sessions. While we as the researchers noticed this, the group members commented on it as well:

*“I think as we were meeting as a group, that if someone was feeling a bit down for some reason, I think the group on the whole was able to help.”*

*“Yes, it made me feel good going and I felt a lot better when I came out of that meeting than when I went in.”*

The group provided a place for renewal of hope. People came to the group expecting to talk about hope. Sometimes they were feeling down or discouraged and found a renewed sense of hope through the support and encouragement of other group members.

#### 4.2.3. Theme 3: Normalizing Experience

Being in the group and listening to each other's stories showed the group members that they were not alone and that other people had experiences and struggles as well. It was interesting to note that when people were asked to share their stories of hope, they often originated in stories of how their hope had been challenged and how they overcame those challenges:

*“Well it showed me that my attitude towards it, that I wasn't way out there on a limb by myself, that there were others that you know, felt the same way”.*

*“Well, I think it's very good to learn about other peoples' problems, Not keep all your own all the time.”*

#### 4.2.4. Theme 4: Developing a More Positive Perspective

Being part of the group and focusing on hope strategies invited participants to imagine or consider a different perspective. Through the group sessions, participants spoke about how they were beginning to see their own situations in a different light. The invitation to consider another perspective frequently encouraged them to think about it from the perspective of a hopeful person:

*“There's nothing wrong with this life here. It's okay. I've got my freedom. I can go shopping. I can do what I want, go out when I want to and yeah. It's very good.”*

*“I've got good eyes…wonderful eyes! My hearing might not be so good but I can sure see! I can walk. I'm not in a wheel chair. I've got two feet that move.”*

Seeing and hearing others hopeful perspectives provided examples of how it was possible to hope in situations that sometimes seemed hopeless. Being in the group sparked a different perspective for some participants. It was freeing to see how others could be hopeful even though they were living with frailties and physical challenges.

#### 4.2.5. Theme 5: Thinking about Hope More Intentionally

An observation that we as researchers made, is that the group participants began to think about hope more intentionally. As the participants reflected on their experience in the group, they talked about how they thought about and noticed hope more intentionally than they ever had, not only in the group but in their daily lives. Frequently they would report on something hopeful they had seen on television or bring in a clipping that reflected hope. As one woman said:

*“Well, I think the group made you think a lot more about the word hope and it just fits into so many places. Probably places that unless somebody spoke about it, that you wouldn't think about.”*

Hope has an individual and a social dimension. Individually, it is that necessary ingredient that enables the participants to cope with their lives as they are. As a result of their life experiences, they know that hope is based on real, possible expectations and not wishful thinking. Hope has helped them to reach out to life's possibilities in the past and continues to do so within the context of their current living situation. Participating in a group focusing on hope revealed its social dimension. Hope, for the participants, was enhanced by sharing experiences of it with each other. Coming to see that they are not alone in their experience, that others will support them when they are down, and that they can support others, all served to spark a different outlook, a different way of seeing. They could better see the positives in their lives and not just focus on the diminishments. They could be more intentional in noticing hope and the good in their lives because another group member had encouraged them to think of something they simply had not thought of before.

## 5. Discussion and Implications for Nursing

Findings from this study suggest that hope is not static. It can increase or decrease depending on situations or circumstances. Health has a major effect on hope for these participants. One said “I suppose when I've been sick. You know I remember from that chair to the bed this might as well have been a hundred miles.” Getting bad news strongly affects hope. One participant recounted being told by medical staff “there is no hope, it's like a body blow.” She adamantly stated, there is “*always hope*” so being told there is no hope is like “closing a door.” “Where there's life, there's hope” is a strongly felt sentiment. This is consistent with Eliott and Olver's [6] analysis that “hope could enable one to endure adversity in life and that, even in adversity, while there is life there is hope” (p. 632).

The participants frequently spoke about ways in which staff and health care professionals fostered their hope. Simple things like greeting them by name (or not) influenced their hope. Nurse-staff interaction is crucial in long-term care. This was reinforced in a study by Haugan [8] in which nursing home patients stressed the need for staff to demonstrate caring attitudes and respect. Indeed the behaviours of staff were critical and were “interpreted as a confirmation of the patient's worthiness or worthlessness” (p. 8). A case study of an 84-year-old woman living in assisted care demonstrated that her hope was increased through specific interventions designed to sustain hope. These interventions focused on providing a caring environment and nurturing ongoing connectedness with others [28].

It was evident that hope has a spiritual quality where the residents acknowledged a trust in something larger, more encompassing. It was reflected in their beliefs that “things will be looked after”… “That's what I know, that I'm okay for today and the good Lord will look after me tomorrow.” “I have faith that whatever happens I am going to be okay.” “My hope is in the Lord.” Yeasting and Jung [29] described a framework for fostering hope which included helping clients to internalize unique hopes through spirituality and finding meaning in life. This suggests that health care professionals have an opportunity to assess residents' spiritual beliefs and how hope might be strengthened through their beliefs. Adults in long-term care are in the last phase of life. Health care professionals can help them reflect back on their lives and assist them in understanding how hope and meaning have been experienced.

Throughout the process of this project, hope was fostered through the experience of being in the group and through being part of this community. Group members clearly identified that, as they listened to each others' stories in the group, they became aware of times when someone was struggling and they took efforts to encourage that person and to offer hope through their caring and concern. The group setting was both the source of expressing one's struggle and making hope more visible through direct strategies used by other group members to encourage and foster hope. There was a sense of connectedness among the group members that was evident within the group but carried on outside the group. Connectedness has been identified as a key ingredient in fostering hope in others [3, 8].

A group setting can provide an important mechanism for nurses and other health care professionals to foster hope. Yeasting and Jung [29] noted that hope is an implicit rather than an explicit idea in practice of family therapists. She explained that “few clinicians have taken up the challenge to articulate specific connections between hope as a theoretical construct and hope as practice” (p. 5). As this project illustrated, a group setting is an ideal place to intentionally use hope in practice by using the group process to introduce hope strategies as the work of the group. While Herth's study [22] found that a hope intervention offered in a group setting with cancer patients served to foster hope, it was conducted with individuals from 21 to 80-year old. Her study suggested that it was possible for hope to be nurtured in dire circumstances (e.g., individuals diagnosed with a recurrence of cancer). Hope and how it is experienced “may be different for older adults than it is for younger adults, which adds important insight into the tailoring hope interventions for this population” [30]. The current study adds a beginning understanding of how a hope intervention in a group of older persons living in a long-term care centre (most of who were living with significant physical health challenges) can facilitate and strengthen the hope of nursing home residents.

Throughout the entire group experience, it was evident that the residents saw hope as acceptance of one's circumstances. They acknowledged that there are aspects of their lives that will not change, but they became aware that hope always remains a possibility. Two participants comments eloquently speak to this: “Well, you certainly hope that things are going to turn out the way that you would like them to. Sometimes there's a shift in what transpires but you try to hang on to that word hope.” “You could hope that other things go a little better, that they satisfy you a little more than just always wishing for something that may happen and may not.” A common sentiment expressed by the participants is that with hope as possibility, there is always something to hope for.

The participants are of a generation that is familiar with difficult circumstances such as war and its accompanying horrors. As one person said “there is no worse than what I went through, the depression…, and significant loss of loved ones.” They came to the group already knowing the importance of hope and hoping. They left the group with that importance reenforced.

As group leaders, it was our observation that while the participants did not describe hope much differently after their group experience, there were some significant differences in our observations during the group process. During these group sessions that specifically focused on hope, participants shared at a much deeper level than they had previously shared in the group. There was a heightened sensitivity to one another's needs and to the group leaders' experiences. Some might interpret this as trust had already been established because this was an ongoing group. On the other hand, the change to this deeper level of sharing in the group coincided with the focus on hope, a fact that suggests that making hope more visible generated reflection on the part of the group participants and a heightened level of incorporating more hopeful thinking in their day-to-day activities. Perhaps another explanation for this can be found in the interaction between the group leaders and the residents. Haugan [8] found that the nurse-patient interaction directly influenced hope, meaning in life, and self-transcendence in nursing home patients suggesting that, in the present study, the group leaders' focus on the experience of older adults being in the group strengthened the “nurse-patient” interaction and thus helped to foster hope.

## 6. Limitations

This study was conducted with women only due to the convenience of access to an existing group. While conducting the study with an established group has many advantages, it would be useful to repeat the intervention with a group that has not met before and with a mixed or all-male group. While it was not the intent of this study to measure levels of hope, it would be useful to repeat this study with other groups of older adults and use a hope scale to assess levels of perceived hope before and after group intervention and to evaluate how increased levels of hope are sustained over time. Further, we recognize that some of the results may have been influenced by the fact that the researchers knew the group participants when the group intervention started and a certain level of trust may have already been established. However, it was our observation, as noted earlier, that it was only specifically the hope-focused sessions during which deeper levels of sharing occurred.

## 7. Conclusions

The findings in this study underscore the central importance of hope in the day-to-day lives of residents in long-term care. Hope is a necessary ingredient for a good quality of life. Combining a focus on hope with a group experience produced a result that was greater than the sum of its parts. It produced a virtuous circle in which focusing on hope added to the depth of sharing and feelings of connectedness amongst the participants. This, in turn, expanded their awareness of hope. The role that this feeling of connectedness plays in the experience of hope in the day-to-day lives of residents has implications for long-term care facilities. Facilities are encouraged to provide programming that includes residents coming together and interacting with each other and using these times for facilitating hope focused activities. This can be achieved through formal programs such as using the hope-focused program described in this study. As staff become aware of the importance of hope both in the lives of their residents and their own lives, it may be possible for hope to be transformed in ways that add to the quality and meaning of life and, in turn, make hope more visible in the day-to-day experiences of living in long-term care.

## Figures and Tables

**Table 1:** Demographic characteristics of study participants.

Age range	Marital status	Children	Educational background	Occupation
75–99	1 never married and 9 widowed	Number of children born by these women 0–8. Four women had lost 1 child.	Grade school through business college and university degree	Nurse
				Ran dairy farm
				Homemaker
				Lab assistant
				Teacher
				Office work
				Medical assistant

**Table 2:** Focusing questions for group sessions.

Group session	Questions to facilitate hope discussions
Session 1: Exploring the concept of hope	What is hope?
	How do you define hope for yourself?

Session 2: Noticing signs of hope	What are the signs of hope that you notice in everyday life?

Session 3: Listening to the voices of hope	What voices of hope have you heard?

Session 4: Looking back at personal experiences of hope	What are your hope stories?

Session 5: Finding hope in unexpected places	Where have you found hope that surprised you?
	Where have you noticed hope that you wouldn't have expected?

Session 6: Borrowing hope. Exploring the concept of borrowing and lending hope	In what ways have you ever given or leant hope to another person?
	Have there been times when you felt that someone gave you hope?

Session 7: Learning to tilt	What are the things that are under your control?
	What are the things that are not under your control?
	What are some of the ways that this situation might turn out better than you expected?
	How might you imagine another perspective?

Session 8: Making one small difference	What can you do this week that would make a small difference?

Session 9: Celebrating hope. Wrap up and celebrate hope	How will you choose to make hope more visible?
